# An Extended Analytical Solution of the Non-Stationary Heat Conduction Problem in Multi-Track Thick-Walled Products during the Additive Manufacturing Process

**DOI:** 10.3390/ma14237291

**Published:** 2021-11-28

**Authors:** Dmitrii Mukin, Ekaterina Valdaytseva, Gleb Turichin, Artur Vildanov

**Affiliations:** World-Class Research Center “Advanced Digital Technologies”, State Marine Technical University, 190121 Saint-Petersburg, Russia; valdaitseva@mail.ru (E.V.); gleb@ltc.ru (G.T.); wildam92@mail.ru (A.V.)

**Keywords:** direct laser deposition, additive manufacturing, analytical modeling, transient temperature field

## Abstract

An analytical model has been developed for calculating three-dimensional transient temperature fields arising in the direct deposition process to study the thermal behavior of multi-track walls with various configurations. The model allows the calculation of all characteristics of the temperature fields (thermal cycles, cooling rates, temperature gradients) in the wall during the direct deposition process at any time. The solution of the non-stationary heat conduction equation for a moving heat source is used to determine the temperature field in the deposited wall, taking into account heat transfer to the environment. The method considers the size of the wall and the substrate, the change in power from layer to layer, the change in the cladding speed, the interpass dwell time (pause time), and the heat source trajectory. Experiments on the deposition of multi-track block samples are carried out, as a result of which the values of the temperatures are obtained at fixed points. The proposed model makes it possible to reproduce temperature fields at various values of the technological process parameters. It is confirmed by comparisons with experimental thermocouple data. The relative difference in the interlayer temperature does not exceed 15%.

## 1. Introduction

The development of additive manufacturing (AM), particularly direct energy deposition (DED) technologies, creates more and more opportunities with the lowest economic and time costs for enterprises of different industries in the manufacture of large-sized, non-standard metal products or parts with a complex shape, as well as in repair and restoration work [1,2,3,4]. In the DED process, a heat source is used to form a melt pool on the surface of a solid metal substrate. The filler material is fed into the melt pool, which causes it to melt. Thus, there is an increase in the volume of the molten material and the formation of the deposited bead. Then, the process is repeated layer-by-layer. There are various direct deposition technologies, differing in the type of heat source used and the filler material used. Technologies using laser and metal powder are well-developed and widespread. The powder is fed into the treatment area by means of protective gas (such as argon), most often either coaxially or multi-jet [5]. A laser, electron beam, and arc can be used when using filler wire. When using a laser or electron beam, the wire is usually fed from the side, which imposes corresponding restrictions on the product shape [6]. The process of using an electric arc as a heat source is similar to fusion welding [7].

The formation of a product layer-by-layer means that the deposited material undergoes quasi-periodic heating and cooling processes, including partial remelting of already formed layers. The resulting non-stationary thermal conditions significantly affect the local microstructure, residual stresses, and deformations, as well as the distribution of defects [8]. Gushchina et al. [9] investigated the effect of the interpass temperature on the Ti-6Al-4V structure in the direct laser deposition (DLD) process. The temperature values varied from approximately 250 to 550 °C. A high interpass temperature corresponds to a lower cooling rate. It leads to the formation of an equilibrium structure, to the elongation values increased by two times, but the microhardness values are underestimated. In [10,11], reduced flow stress was found for material obtained by AM (selective laser melting (SLM), electron beam melting (EBM), and DED) compared to wrought material in compression tests at high temperatures. However, it was found in [12] that the Young’s modulus of the DLD-processed Ti-6Al-4V alloy is approximately 46% higher at 500 °C than that of the wrought alloy. In the study [13], deposited Ti-6Al-4V samples showed low values of relative elongation and uneven grain size across the sample width. On the other hand, when a wobbling or oscillating laser beam is used, an even distribution of grain sizes, a uniform distribution of hardness, and a high value of mechanical properties are already observed. This allows the use of such products without additional heat treatment. There are also attempts to develop maps of the microstructure evolution based on experimental results, which showed different areas of microstructures depending on the cooling rate and repeated thermal cycles [14,15]. In the AM process, defects are also encountered, such as non-fusion, the main cause of which is insufficient heat source energy while simultaneously feeding an excess amount of filler material into the melt pool [16]. The incorrect choice of the mode can also lead to the formation of cracking and delamination [8]. At the same time, the above effects can be combated by optimizing the process, which includes strict control of operating parameters, the presence or absence of preheating, control of the cooling rate, and the choice of a scanning strategy. Thus, the problem of determining transient temperature fields is not new, but it is still relevant. Experimental determination of temperature fields, cooling rates, as well as related regularities is time-consuming and costly due to the large number of technological parameters in the process. Generally, temperature measurement is carried out by a contact method based on thermocouples and a non-contact method based on infrared thermography [17,18,19]. However, infrared thermography does not allow direct measurements of the object’s surface temperature. The accuracy of determining the surface temperature is limited by the unknown emissivity ε. Moreover, emissivity is not constant but depends on many factors, such as the material, surface temperature, surface roughness, viewing angle, and presence of oxide films [20,21]. Thermocouples can significantly improve the accuracy of measurements. For this reason, they are used both for measuring temperature and for calibrating the thermal camera.

Another way to determine three-dimensional temperature fields is to use different methods of mathematical modeling. One of the most common methods for calculating transient temperature fields in the AM process is the finite element method (FEM) [22,23]. The material deposition in the DED process is often modeled using quiet or inactive elements, which are activated as the added filler material solidifies [24,25]. In the quiet approach, the elements are present in the calculations but are assigned special properties. For this reason, such elements do not affect the result. In the inactive element approach, elements are not included in the calculations until the corresponding material has been added. Michaleris [26] proposed a numerical model using the hybrid quiet/inactive element method. This approach assumes that the elements are initially inactive and then switch to quiet mode. As a result, equivalent results can be obtained with less computation time. Peyre et al. [27] developed a three-stage DED model, taking into account the powder temperature and the deposited bead geometry. Before solving the heat transfer problem using FEM, the layer height is calculated based on the law of conservation of mass and energy, and the layer width is calculated by the iterative method. Heigel et al. [28] compared the results of modeling temperature fields under various convection conditions. According to the results, the forced convection model gives the best agreement with the experimental data as compared to the free convection model. Chiumenti et al. [29] showed that heat loss occurs mainly through thermal radiation. This effect cannot be neglected but it can be replaced by a temperature-dependent convective heat transfer coefficient that will exceed the free convection coefficient. A great deal of research is aimed at reducing the calculation time. A widespread approach is that the material is added either in parts of a layer, in whole layers at once, or in several layers at once [23,25]. Denlinger [30] introduced layer-by-layer coarsening of the mesh at a certain distance from the heat source. Patil, Pal et al. [31] used a fine mesh only locally near the heat source. With these strategies, it is possible to reduce the number of elements without losing the high accuracy of the solution.

There are also numerical approaches that take into account convective heat transfer and mixing in the melt pool [32]. Using the control volume method, Manvatkar et al. [33] demonstrated that neglecting the liquid metal flow leads to an overestimation of the temperature field—in particular, peak values of the temperatures. This result was confirmed by Gan et al. [34], who also indicated that the Marangoni convection causes mixing in the molten pool, as a result of which the distribution of the composition evens out rapidly to a uniform value. This must be taken into account when solving the crystallization problem.

There is also an analytical approach to determining temperature fields [35,36,37]. However, such works have several characteristic features. First, heat transfer to the environment is not taken into account, which, as is known, has a significant effect on temperature [29]. In addition, the adiabatic boundary of surfaces (or convective heat transfer) is not specified in the calculations, or the adiabatic boundary is specified only on some surfaces. The physical interpretation of the adiabatic boundary is that the heat transferred to the body remains inside the body. The case in the absence of an adiabatic boundary corresponds to a body that is infinite in all directions; the case with adiabatic boundaries on some surfaces corresponds to a body with an infinite size in the direction where there is no adiabatic boundary. From a physical point of view, it corresponds to a different problem statement. In addition, no works have been found in the literature that would correctly apply the analytical solution to the determination of transient temperature fields over long periods.

The objective of this work is to develop a simple and fast but, at the same time, reliable method for calculating transient three-dimensional temperature fields in the AM process of multi-track thick-walled products. This work is a continuation of a previous work [38], where the heat conduction problem was solved for single-pass thin-walled products. The developed calculation scheme assumes its further inclusion in the optimization system for selecting the technological parameters. It presupposes the processes of multiple direct calculation (direct problem). As a consequence, the issue of computation time is fundamental. Analytical methods are inferior in accuracy to numerical methods, but they make it possible to directly determine the relationship between individual parameters of a mode or a group of parameters. In such conditions, high accuracy of the calculation is not a fundamental point, since the process is extremely complex and some assumptions will have to be made in any case. In addition, due to the lack of accurate values of the thermophysical properties of materials in high-temperature regions, the calculation accuracy using any method is, in principle, limited. Thus, in decision support systems for technologists or engineers, the issue of quickly determining relationships comes to the fore. As a result of using the extended solution, it becomes possible to study the degree of influence of the process parameters and different heat source trajectories on the temperature distribution and cooling rate in the product. From a practical point of view, it is applicable because maintaining stable temperatures and melt pool sizes is one of the key means of controlling the process stability.

## 2. Methods and Model Description

### 2.1. Problem Statement

The following physical assumptions were made in the heat transfer model:

- The thermophysical properties of the filler material and substrate are constant and temperature-independent;
- Heat flux distribution of the heat source *q_h_* is presented as a surface normally distributed heat source;
- Heat transfer occurs according to Newton’s law;
- Phase transition is not considered;
- The effect of convection of liquid metal is neglected.

Transient heat transfer throughout the model is determined by a three-dimensional linear heat conduction equation in a Cartesian coordinate system with appropriate boundary conditions. It is worth considering that heat is released into the environment from the wall side surfaces under conditions of convective heat transfer. Then, convective heat transfer can be represented as a heat sink $q_{sc}=-\frac{\alpha T}{W}-\frac{\alpha T}{W}=-\frac{2\alpha T}{W}$, where α—surface heat transfer coefficient, and *W*—wall width. This approach considers only the decrease in the average temperature in the section, and does not consider the temperature unevenness along the wall width. The heat conduction equation is expressed as:

(1) $$\frac{\lambda }{c\rho }\left(\frac{\partial^{2}T}{\partial x^{2}}+\frac{\partial^{2}T}{\partial y^{2}}+\frac{\partial^{2}T}{\partial z^{2}}\right)-\frac{2\alpha }{c\rho W}T=\frac{\partial T}{\partial t}$$

where *λ*—thermal conductivity, *ρ*—density, and *c*—specific heat capacity.

The initial temperature distribution of the substrate and the wall at time *t* = 0 is equal to the ambient temperature *T*_0_.

Boundary conditions on the front surface of the computational domain are as follows:

(2) $$-\lambda \frac{\partial T}{\partial n}=q_{h}(x,y)$$

where *q_h_*(*x, y*) is the heat flux density.

The adiabatic boundary is set on other surfaces of the wall and substrate where there are no heat sources.

### 2.2. Analytical Model of Transient Heat Transfer

**Figure 1:**
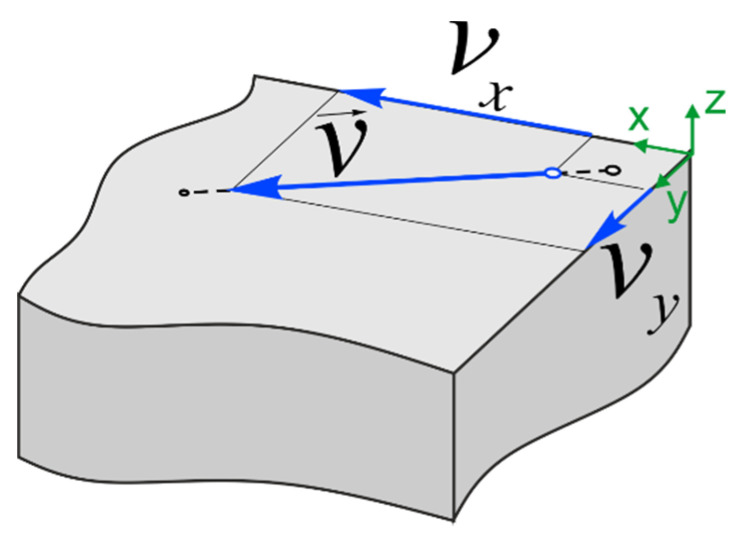
Cladding speed vector projection on the *x*- and *y*-axes.

The solution to Equation (1) can be obtained using the Green’s function method for the heat equation. Then, the temperature increment at an arbitrary point with coordinates *x*, *y*, *z* at any time *t* from an elementary point source (moving with speed *v*) that acted at time *t*′ on the surface of a semi-infinite body in a stationary coordinate system is equal to *dT*:

(3) $$dT(x,y,z,t,t^{\prime })=\frac{2q\;dt^{\prime }}{c\rho {\left[4\pi a(t-t^{\prime })\right]}^{3/2}}\exp\left(-\frac{{\left[x-v_{x}t^{\prime }\right]}^{2}+{\left[y-v_{y}t^{\prime }\right]}^{2}+z^{2}}{4a(t-t^{\prime })}-b(t-t^{\prime })\right)$$

where *q*—point heat source power, *a*—thermal diffusivity, *v*_x_—the projection of the heat source speed *v* onto the *x*-axis (Figure 1), *v*_y_—the projection of the heat source speed *v* onto the *y*-axis, and $b=\frac{2\alpha }{c\rho W}$—coefficient of heat loss.

The solution for a moving point source can be obtained by taking the integral over the source action time *t*′ from *t*_1_ to *t*_2_ (the source started acting at time *t*_1_ and finished acting at time *t*_2_):

(4) $$\begin{array}{l} \Delta T(x,y,z,t,t_{1},t_{2})=\frac{2q}{4\pi \lambda R}\frac{1}{2}\exp\left(-\frac{v_{x}\left(x-v_{x}t\right)}{2a}-\frac{v_{y}\left(y-v_{y}t\right)}{2a}\right)\times \\ \times \{[\exp\left(-\frac{Rv_{xy}B}{2a}\right)\cdot \Phi^{*}\left(\frac{-R}{2\sqrt{a(t-t_{2})}}+\frac{v_{xy}B\sqrt{\left(t-t_{2}\right)}}{2\sqrt{a}}\right)-\exp\left(\frac{Rv_{xy}B}{2a}\right)\Phi^{*}\left(\frac{R}{2\sqrt{a(t-t_{2})}}+\frac{v_{xy}B\sqrt{\left(t-t_{2}\right)}}{2\sqrt{a}}\right)]-\\ -[\exp\left(-\frac{Rv_{xy}B}{2a}\right)\cdot \Phi^{*}\left(\frac{-R}{2\sqrt{a(t-t_{1})}}+\frac{v_{xy}B\sqrt{\left(t-t_{1}\right)}}{2\sqrt{a}}\right)-\exp\left(\frac{Rv_{xy}B}{2a}\right)\Phi^{*}\left(\frac{R}{2\sqrt{a(t-t_{1})}}+\frac{v_{xy}B\sqrt{\left(t-t_{1}\right)}}{2\sqrt{a}}\right)]\}, \end{array}$$

where $v_{xy}=\sqrt{v_{x}{}^{2}+v_{y}{}^{2}}$, $R=\sqrt{{\left(x-v_{x}t\right)}^{2}+{\left(y-v_{y}t\right)}^{2}+z^{2}}$—distance from the heat source to the considered point, $B=\sqrt{1+\frac{4ba}{v_{xy}{}^{2}}}$, $\Phi^{*}(u)=1-\frac{2}{\sqrt{\pi }}\int_{0}^{u}e^{-u^{2}}du$, and *t* > *t*_2_ ≥ *t*_1_ ≥ 0.

**Figure 2:**
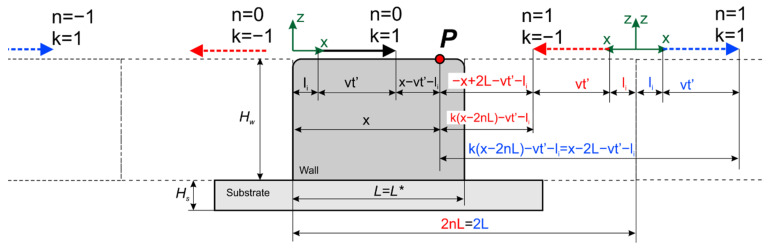
Scheme of introducing imaginary sources to take into account the limited size of the wall in length or width.

**Figure 3:**
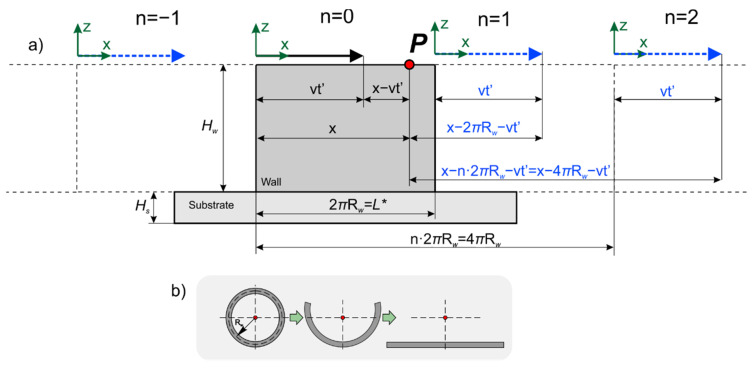
Scheme for taking into account the limited size of a closed wall along the length: (**a**) scheme of introducing imaginary sources; (**b**) scheme of unwrapping of a closed wall (cylindrical wall).

Let us pass from the solution for a semi-infinite body to the solution for a bounded body. In order to satisfy homogeneous boundary conditions of the second kind in the form of the absence of heat flow through the wall boundaries, it is necessary to continue the function, defining the heat source as an even function relative to the body boundaries. Then, the resulting even function is continued periodically on the entire numeric axis. The physical interpretation of this action consists of the introduction of imaginary heat sources symmetrically relative to the body’s boundaries. In this case, the planes of symmetry are the planes *x* = 0, *x* = L^*^, where L^*^ is the wall length, from the planes *z* = 0 and *z* = H, where H is the wall height (sum of substrate height *H_s_* and wall height *H_w_*), and also from the side wall boundaries *y* = 0, *y* = *W*. Figure 2 and Figure 3 show the schemes of introducing imaginary sources along the *x*-axis and *y*-axis for a thick wall and a closed wall. The scheme for a closed wall (in particular, a cylindrical wall) can be obtained by unwrapping the wall around one of its generatrices (Figure 3b). The temperature field is calculated at an arbitrary point *P*.

Then, the temperature field in the deposited wall is defined as an infinite series of corresponding solutions for an unbounded semi-infinite body (in this case, the source is usually distributed over the computational domain surface):

(5) $$\begin{array}{l} dT(x,y,z,xs,ys,t,t_{1},t_{2})=\sum_{p=-\infty }^{+\infty }\sum_{j=-\infty }^{+\infty }\sum_{n=-\infty }^{+\infty }\sum_{kx}^{}\sum_{ky}\frac{2q_{h}(xs,ys)dxs\;dys}{4\pi \lambda R}\frac{1}{2}\exp\left(-\frac{v_{x}\left(X-v_{y}t-xs\right)}{2a}-\frac{v_{y}\left(Y-v_{x}t-xs\right)}{2a}\right)\times \\ \times \{[\exp\left(-\;\frac{Rv_{xy}B}{2a}\right)\cdot \Phi^{*}\left(\frac{-R}{2\sqrt{a(t-t_{2})}}+\frac{v_{xy}B\sqrt{\left(t-t_{2}\right)}}{2\sqrt{a}}\right)-\exp\left(\frac{Rv_{xy}B}{2a}\right)\cdot \Phi^{*}\left(\frac{R}{2\sqrt{a(t-t_{2})}}+\frac{v_{xy}B\sqrt{\left(t-t_{2}\right)}}{2\sqrt{a}}\right)]-\\ -[\exp\left(-\;\frac{Rv_{xy}B}{2a}\right)\cdot \Phi^{*}\left(\frac{-R}{2\sqrt{a(t-t_{1})}}+\frac{v_{xy}B\sqrt{\left(t-t_{1}\right)}}{2\sqrt{a}}\right)-\exp\left(\frac{Rv_{xy}B}{2a}\right)\cdot \Phi^{*}\left(\frac{R}{2\sqrt{a(t-t_{1})}}+\frac{v_{xy}B\sqrt{\left(t-t_{1}\right)}}{2\sqrt{a}}\right)]\}, \end{array}$$

where $R=\sqrt{{\left(X-v_{x}t-xs\right)}^{2}+{\left(Y-v_{y}t-ys\right)}^{2}+{\left(z+2pH\right)}^{2}}$, *xs*, *ys*—the coordinates of the point source in the coordinate system associated with the source, $X=k_{x}(x-2nL^{*})-l_{x}$—for the case of a single wall, $X=k(x-nL^{*})$—for the case of a closed wall, $Y=k_{y}(y-2jW)-l_{y}$, *k* = −1, 1—for the case of a single wall, *k* = 1—for the case of a closed wall, L* = L—for the case of a single wall, and L* = 2πR_w_—for the case of a closed wall. Summation over *n* considers the limited length, while for summation over *j* and *p*—over width and height, respectively.

By shifting the origin for each pass in the AM process, it is possible to set the times *t*_1_ and *t*_2_ in such a way that *t*_1_ = 0 always, and $t_{2}^{}=\;\left\{ \begin{array}{l} t-o(t),if\;t\leq \frac{L^{*}}{v};\\ \frac{L^{*}}{v},\;\;\;\;\;\;\;\;if\;t>\frac{L^{*}}{v}; \end{array}\right.$. In this case, *t*_1_ and *t*_2_ are not arguments to the *dT* function.

If the source acts over an area with radius *R*_b_, then it is necessary to integrate Equation (5) over this area to obtain the temperature field. Then, the temperature Δ*T_preh_*(*x,y,z,t,t*_1_,*t*_2_) can be obtained as:

(6) $$\Delta T_{preh}(x,y,z,t,t_{1},t_{2})=\int_{-Rb}^{+Rb}\int_{-Rb}^{+Rb}dT(x,y,z,xs,ys,t,t_{1},t_{2})$$

The number of series terms in Equation (5), which must be taken into account in the calculations, depends on two aspects: the value of the considered heating or cooling time *t* in the DLD process, and the value of the thermal diffusivity of the material *a*. Thus, the number of series terms is directly proportional to $\sqrt{4at}$.

It is worth noting that the variable *R* in Equation (5) can have a large value in modulus. This leads to the fact that in the product $\exp\left(\frac{Rv_{xy}B}{2a}\right)\cdot \Phi^{*}\left(\frac{R}{2\sqrt{a(t-t_{1,2})}}+\frac{v_{xy}B\sqrt{t-t_{1,2}}}{2\sqrt{a}}\right)$ the multiplier $\exp\left(\frac{Rv_{xy}B}{2a}\right)$ tends to be a large value, which is taken equal to infinity (infinitely large value). This result occurs when using mathematical and computational software in the calculation process. The multiplier $\Phi^{*}\left(\frac{R}{2\sqrt{a(t-t_{1,2})}}+\frac{v_{xy}B\sqrt{t-t_{1,2}}}{2\sqrt{a}}\right)$ tends toward 0 (infinitesimal value). Consequently, an indeterminate form of the type (0 × ∞) arises in Equation (5).

For the evaluation of the indeterminate form, let us use, for example, the known approximation of the error function erf (*x*) using elementary functions 7.1.26 [39]:

(7) $$\mathrm{erf}(x)=1-\left(a_{1}k+a_{2}k^{2}+\ldots +a_{5}k^{5}\right)e^{-x^{2}}+\varepsilon (x),$$

where $k=\frac{1}{1+px}$, $\left|\varepsilon (x)\right|\leq 1.5\times 10^{-7}$, $x=\frac{R}{2\sqrt{a(t-t_{1,2})}}+\frac{v_{xy}B\sqrt{t-t_{1,2}}}{2\sqrt{a}}$, *p* = 0.3275911, *a*_1_ = 0.254829592, *a*_2_ = −0.284496736, *a*_3_ = 1.421413741, *a*_4_ = −1.453152027, and *a*_5_ = 1.061405429.

Then, it is easy to eliminate the arising indeterminate form using this replacement and performing elementary transformations.

### 2.3. Influence of the Substrate on the Temperature Field

At the initial stage of the deposition process, the conditions for the formation of layers differ as their number increases. The heat accumulation in the wall during the initial deposition period causes different heat removal conditions. A steady state of the process is achieved when the wall temperature stops rising, and the conditions for heat removal become constant. At the same time, the conditions for the formation of each subsequent layer, all other factors being equal, do not differ from the conditions for the formation of the previous layer.

**Figure 4:**
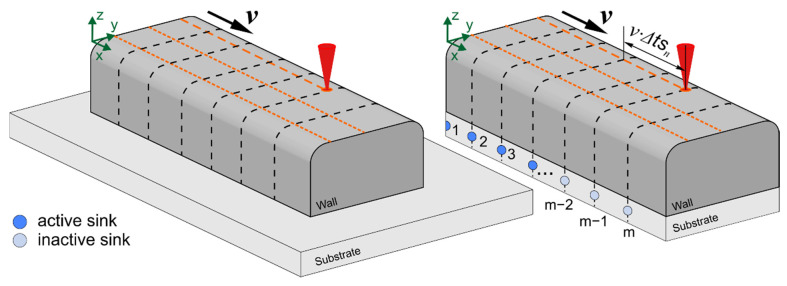
Scheme of the introduction of heat sinks (the sinks are shown for the current pass).

It is convenient to calculate temperature fields using the Green’s function method for bodies of simple geometric shape. The shape of the wall deposited onto the substrate is not simple but rather T-shaped. In this regard, several approximate computational schemes for calculating temperature fields were considered. According to the first scheme, the side parts of a substrate that are outside the wall construction area are removed (Figure 4). To take into account the removed mass of the substrate, heat sinks of the corresponding energy are introduced. One of the variants of this approach is described in [38].

The second approach consists in replacing the real horizontal substrate with a vertical substrate (rib), the section of which coincides with the wall section. It should be borne in mind that the masses of these substrates must be equivalent to each other. However, this approach is applicable only when the wall width exceeds the thickness of the substrate by two times or more. This is due to the fact that the heat removal conditions change with a smaller wall width.

The third approach is a combination of the first two. In this case, the vertical substrate height is half the horizontal substrate width. The remaining removed part of the substrate mass is taken into account by introducing heat sinks.

The temperature increment in the equilibrium state after the next pass is

(8) $$\Delta T=\frac{Q}{cm}=\frac{Q_{h}\eta \frac{L^{\prime }}{v}}{c\rho V_{s}+c\rho V_{w}}$$

where *L*′—the trajectory length of the heat source on the current pass, *V_s_*—the substrate volume, and *V*_w_—the wall volume.

Based on the condition that the increment in the wall temperature in the absence of a substrate corresponds to the increment in the wall temperature in the presence of a substrate, it is possible to determine the total energy of the sinks at each pass:

(9) $$E_{n}=Q_{h}\;\eta \frac{L^{\prime }}{v}\left(1-\frac{c\rho {V_{s}}^{\prime }+c\rho V_{w}(n)}{c\rho V_{s}+c\rho V_{w}(n)}\right)$$

where *V_s_*′—truncated substrate volume, and *V*_w_(*n*)—wall volume on the *n*-th passage.

The power of each fixed sink is determined based on the time of their action *ts_n_* and the total energy of all sinks *E_n_*. Let us take into account that the power of each sink is not constant, but decreases exponentially in time according to the following law:

(10) $$q_{s}^{n}(t)=Q_{s}^{n}\;\exp\left(-\frac{4t}{ts_{n}}\right)$$

The action time of the sinks is proportional to the distribution time of uneven temperature; that is, $ts_{n}\sim \frac{R_{n}{}^{2}}{4a}$, where *R_n_* is the characteristic size of the deposition wall after *n* passes. The maximum power of each sink can be determined using the expression

(11) $$Q_{s}^{n}=\frac{E_{n}^{}}{2\cdot m\cdot \int_{0}^{ts_{n}}\exp\left(-\frac{4t}{ts_{n}}\right)dt}$$

where *m*—number of sinks.

The inertia of the heat transfer process in the wall is taken into account due to the introduction of the delay time $\Delta ts_{n}$ of the action of the sinks in relation to the actual heat source action (see Figure 4), while $\Delta ts_{n}\sim \frac{H_{n}^{2}}{4a}$.

To obtain the equation of the temperature field as a result of the action of sinks, suppose *v* = 0 in Equation (3):

(12) $$\Delta T_{sk}(x,y,z,t,t_{1},t_{2})=\sum_{i=0}^{m-1}\;\sum_{u=-1,1}^{}\;\sum_{p=-\infty }^{+\infty }\;\sum_{j=-\infty }^{+\infty }\sum_{n=-\infty }^{+\infty }\sum_{kx}^{}\;\frac{2}{c\rho {\left(4\pi a\right)}^{3/2}}\int_{t1}^{t2}\frac{q_{s}^{n}(t^{\prime }-t_{1})\;dt^{\prime }}{{\left(t-t^{\prime }\right)}^{3/2}}\cdot \exp\left(-\frac{R{\left(x,y,z\right)}^{2}}{4a(t-t^{\prime })}-b(t-t^{\prime })\right)$$

where $R=\sqrt{{\left(k_{x}(x-2nL^{*})-\left[\frac{i}{m}L^{*}\right]\right)}^{2}+{\left(y+jW\right)}^{2}+{\left(z-u\left(H-\frac{H_{s}}{2}\right)+2pH\right)}^{2}}$,

$$t_{1}^{i}=\;\left\{ \begin{array}{l} t-o(t),\;\;\;\;\;\;\;\;\;\;if\;t\leq \frac{i}{m}\frac{L^{*}}{v}+\Delta ts;\\ \frac{i}{m}\frac{L^{*}}{v}+\Delta ts,\;\;\;if\;t>\frac{i}{m}\frac{L^{*}}{v}+\Delta ts; \end{array}\right.\;t_{2}^{i}=\;\left\{ \begin{array}{l} t-o(t),\;\;\;\;\;if\;t\leq ts+\Delta ts;\\ ts+\Delta ts,\;\;\;if\;t>ts+\Delta ts; \end{array}\right.$$

Another way to take into account the removed mass of the substrate is to introduce a volumetric sink (*m* = 1), the temperature field of which can be determined by the following equation:

(13) $$\Delta T_{sk}(x,y,z,t,t_{1},t_{2})=\int_{t1}^{t2}\frac{2q_{s}^{n}(t^{\prime }-t)dt^{\prime }}{c\rho \left(V_{s}+V_{w}(n)\right)}\exp\left(-b(t-t^{\prime })\right)$$

The total temperature field *T_n_* after the deposition of the *n*-th number of layers can be represented as a superposition of temperature fields from the action of heat sources and the action of sinks at each pass. In this case, the process parameters (power, cladding speed, trajectory, pause time) can differ at each pass. Then, the heating temperature can be calculated using the following equation:

(14) $$T_{n}(x,y,z,t)=T_{0}+\sum_{i=1}^{n}\;[\Delta T_{preh}\left(x,y,z,t+\sum_{j=1}^{i}\;\left[\frac{L_{j}{}^{*}}{v_{j}}+t_{pause\;\;j}\right]\right)-\Delta T_{sk}\left(x,y,z,t+\sum_{j=1}^{i}\;\left[\frac{L_{j}{}^{*}}{v_{j}}+t_{pause\;\;j}\;\right]\right)]$$

where *t*_pause_—the pause time between passes, index *i* = 1 corresponds to the last pass, and the index *i* = *n* corresponds to the first pass.

**Figure 5:**
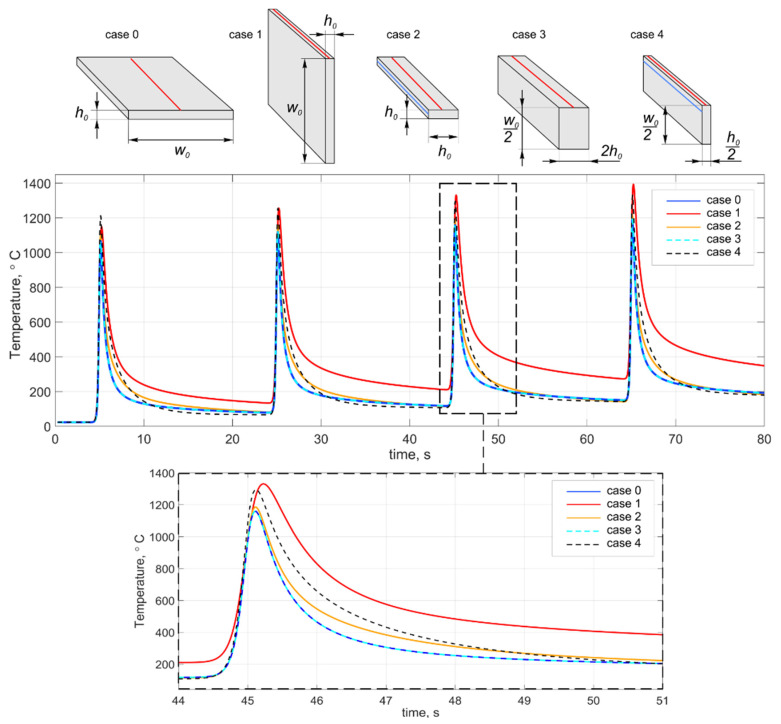
Calculated temperature cycles when replacing an actual substrate with an approximate one (using various approximate calculation schemes).

It should be noted that the above approaches to determining the temperature field are applicable since the difference in heat removal condition is insignificant. As a result, it can be ignored. Let us demonstrate this by the example of the repeated heating of a steel substrate with a size of 200 × 100 × 10 mm (length × width × height) in its middle part. The calculation results are compared with the results of using approximate calculation schemes. Case 0 corresponds to the heating of the horizontal substrate in its middle part; case 1 is a vertical substrate (rib) with a wall width *w*_0_ = *h*_0_; case 2 is a truncated substrate with a wall width *w*_0_ = *h*_0_; case 3 is a vertical substrate with a wall width *w*_0_ = 2*h*_0_; case 4 is a vertical substrate with a wall width *w*_0_ = *h*_0_/2 and a height equal to the half-width of the substrate (Figure 5).

The difference between the curves for cases 2, 3, and 4 compared to the heating of the real substrate (case 0) is insignificant. As mentioned earlier, case 1 has reduced heat removal. The temperature deviation is observed upward at a short time interval for cases 2 and 4 after the passage of the temperature peak. The smaller the wall width, the greater the difference between the actual temperature and the calculated one. However, all curves converge to the same value. Moreover, this effect is observed only at the initial deposition stage, when the wall height is insignificant in comparison with the substrate thickness. The curves completely coincide for the wall width *w* ≥ 2*h*. The real wall will increasingly approach the approximate calculation scheme with an increase in the number of layers. Thus, in this work, we will use a calculation scheme with the replacement of the horizontal substrate by a vertical one at *w* ≥ 2*h* and a calculation scheme with the replacement of the horizontal substrate by a vertical one with a height equal to the half-width of the substrate and heat sinks at *w* < 2*h*.

**Figure 6:**
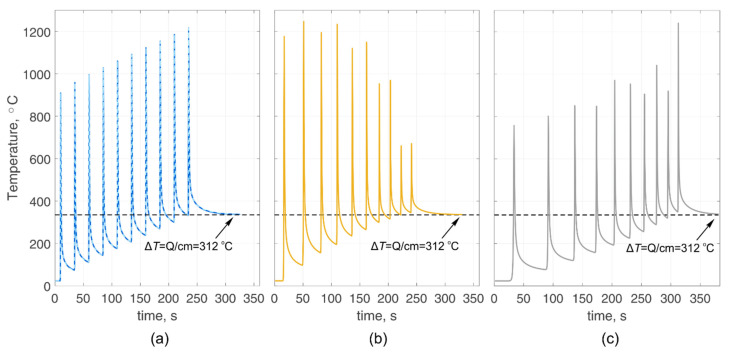
Calculated temperature cycles for arbitrary different modes: (**a**) mode 1 (the actual substrate and the truncated substrate); (**b**) mode 2; (**c**) mode 3.

Let us show that the proposed calculation method also satisfies the law of conservation of energy. Consider several arbitrary modes (10 passes) of heating a body with different powers (pure powers), heat source movement speeds, and pause times between passes (Table 1). However, the total input amount of heat energy remains the same for all cases. The heating body is a steel substrate and a truncated substrate (similar to case 0 and case 2). The truncated substrate width is equal to the half-width of the actual substrate. In this case, heat transfer to the environment is absent, so we will consider the completely adiabatic boundaries of all substrate surfaces. The choice of arbitrary modes will make it possible to draw conclusions about the independence of the result from the specific parameters of the mode. The calculated results are compared with the steady-state temperature (Figure 6) obtained from the equation Q=cmΔT.

Despite the different heating modes (the total invested thermal energy is the same), the body temperature in the calculations has always tended to its equilibrium state at a given energy value. Thus, the coincidence of the temperatures of all curves (in the absence of heat exchange with the environment) indicates the consistency and adequacy of the developed method for calculating non-stationary temperature fields.

## 3. Materials and Experimental Procedure

Powder of titanium alloy Ti-6Al-4V was used as a filler material in the direct laser deposition (DLD) process. Multi-track block samples were produced using a robotic DLD complex based on an IPG fiber laser. The complex included a six-axis robot and a two-axis positioner manufactured by Fanuc. The laser system had a maximum power of 3000 W. The filler powder was fed through a four-jet nozzle coaxially to the laser beam by argon flow. The deposition process was carried out in a sealed chamber with an argon atmosphere.

A multi-track wall consisting of 137 layers was deposited onto a rolled Ti-6Al-4V substrate. The substrate size was 105 × 40 × 8 mm (length × width × height). The deposition wall size was approximately 67 × 14 × 110 mm. Each layer consisted of seven beads. Thus, seven passes formed one deposited layer with a length of 67 mm. The distance between the centers of adjacent beads was 1.67 mm. All passes were carried out in one direction. The vertical step size was 0.8 mm. The process parameters are shown in Table 2. Three samples were obtained with different pause times between passes (interpass dwell time), which were 0, 10, and 25 s.

**Figure 7:**
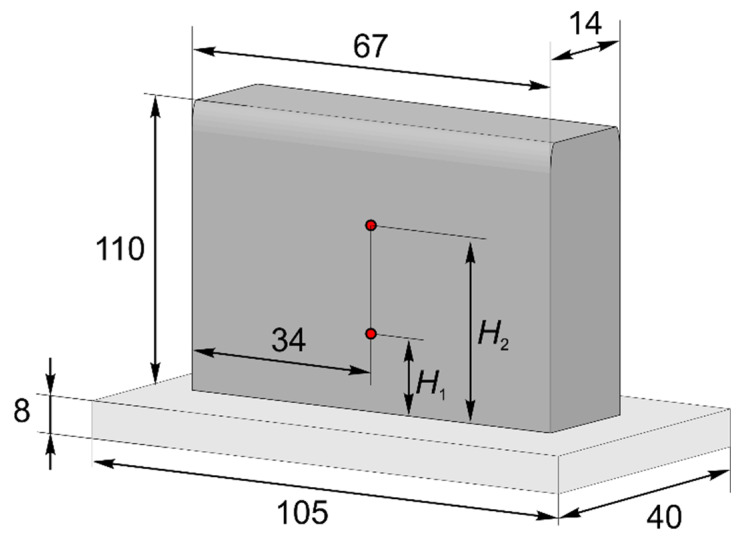
The size of the deposited sample and the location of the thermocouple attachment point.

Temperature measurements were carried out using tungsten/rhenium-alloy thermocouples (Tungsten 95%/Rhenium 5%—Tungsten 80%/Rhenium 20%), the closest analogue of which is a type C thermocouple. Thermocouples were installed on the edge of the upper wall surface (at various distances from the substrate). Then, the thermocouple was fused to the wall, as a result of cladding the next layer. The location of the thermocouple attachment point is shown in Figure 7. The readings of the tungsten/rhenium-alloy thermocouple were previously calibrated using a type K thermocouple in the temperature range of 20–800 °C.

## 4. Results and Discussion

The thermocouple data were used to verify the developed transient heat transfer model. For each sample, two thermocouples were installed at different distances from the substrate. The position of the thermocouples is shown in Figure 7. For mode 1—*H*_1_ = 12 mm (15 layers), *H*_2_ = 78 mm (96 layers); for mode 2—*H*_1_ = 10 mm (12 layers), *H*_2_ = 73 mm (91 layers); for mode 3—*H*_1_ = 12 mm (15 layers), *H*_2_ = 73 mm (91 layers). The modeling parameters were taken according to Table 2. The calculations used the absorption coefficient of laser radiation equal to 0.35, the convective heat transfer coefficient for the mode without a pause was 70 W·K^−1^·m^−2^, and that for the modes with a pause was 35 W·K^−1^·m^−2^. The values of the thermophysical properties were taken from [40] and were adopted as constant, and averaged in the temperature interval between the interlayer temperature of the previous layer and the melting point.

**Figure 8:**
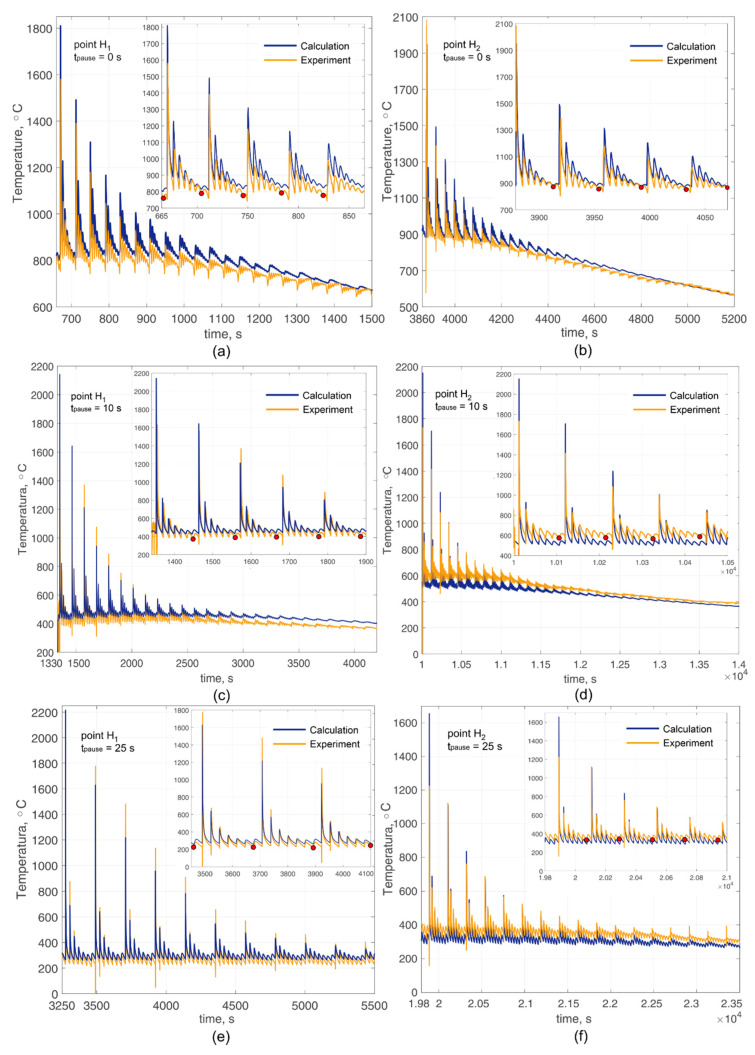
Comparison of calculated and experimental thermal cycles during deposition of the multi-track wall: (**a**) point *H*_1_, pause time is 0 s; (**b**) point *H*_2_, pause time is 0 s; (**c**) point *H*_1_, pause time is 10 s; (**d**) point *H*_2_, pause time is 10 s; (**e**) point *H*_1_, pause time is 25 s; (**f**) point *H*_2_, pause time is 25 s.

Figure 8 shows thermal cycles obtained using thermocouples (point *H*_1_ and *H*_2_), as well as calculation cycles for modes with a pause time between passes of 0, 10, and 25 s. However, it is worth clarifying that a pause of 0 s (as with 10 and 25 s) is a pause that is set in the control program of the robot. The actual pause time was more than 0 (more than 10 and 25 s) since the robot needs a certain time to return from the point of the end of the trajectory to the point of the beginning of the trajectory.

Thermocouples were installed at the deposition of 15th layer (point *H*_1_) and 96th layer (point *H*_2_) for the mode without a pause. For point *H*_1_, the thermocouple readings were considered from the moment the deposition of the 16th layer begins (Figure 8a), which corresponds to 106 passes. For point *H*_2_, the thermocouple readings were considered from the moment the deposition of the 97th layer begins (Figure 8b), which corresponds to 673 passes. Similarly, data were obtained in modes with a pause of 10 and 25 s. However, with a pause of 10 s, thermocouples were installed during deposition of 12 layers and 91 layers, and thermocouples were installed during deposition of 15 layers and 91 layers with a pause of 25 s.

**Figure 9:**
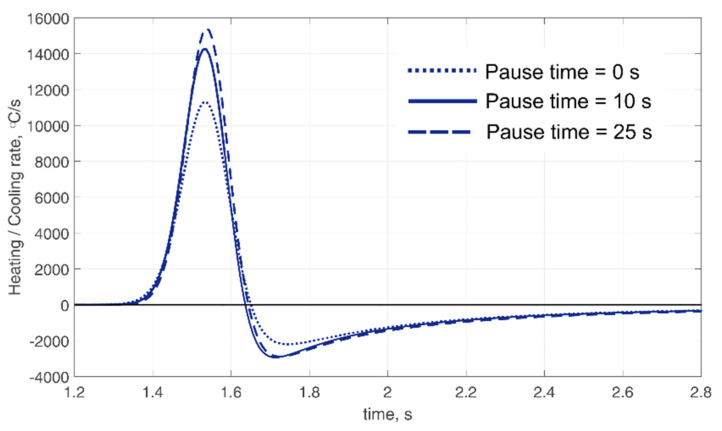
Calculated cooling rates during deposition of the 106th pass (outer bead).

The decrease in the temperature of the fixed points of the wall can be observed over time with a pause time between passes of 0 s (Figure 8a,b). A less intense decrease in temperature is also observed with a pause of 10 s (Figure 8c,d). However, the temperature of the fixed points does not decrease with a pause of 25 s but remains constant (Figure 8e,f), which is completely consistent with the experimental results. A decrease in temperature at a fixed point, as in the case of a mode without a pause, indicates high temperature gradients along with the wall height. On the contrary, in the case of a pause of 25 s, when the temperature hardly changes, the temperature gradient along the height is almost absent. Knowing the change in temperature over time, heating and cooling rates can be found. Figure 9 shows the calculated cooling rates in the near-surface layer of the wall (point *H*_1_) during the deposition of 106th pass (16th layer) at different pause times. The highest cooling rate is observed in the mode with the longest pause. Thus, the longer the pause time, the faster the heating and cooling rate. This is directly related to the value of the interpass or interlayer temperature. At low temperatures, the heat removal process proceeds faster. Cooling rates may differ by 30% according to calculations.

**Figure 10:**
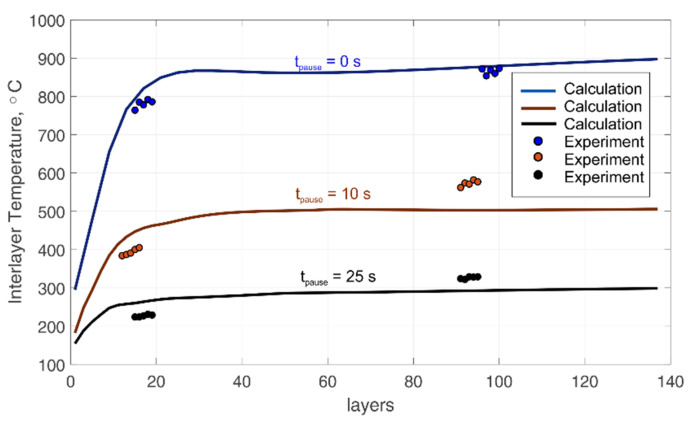
Change in interlayer temperature depending on the pause time between passes.

Let us consider the change in the interlayer temperature depending on the pause time between passes. The temperature of the first five layers after attaching the thermocouple was used to fix the interlayer temperature since the interlayer temperature is actually the wall surface temperature. Moreover, at a shallow depth from the surface, the temperature has time to equalize. The temperature values that were used to determine the interlayer temperature are highlighted with red dots on thermal cycles (see Figure 8a–f). In this case, the temperature values were used after the sixth pass, since, after the seventh pass, outliers are observed. Therefore, these points are ignored. Figure 10 shows the calculated curves of the interlayer temperature depending on the number of layers and the pause time between passes with plotted experimental points.

The temperature is significantly higher when depositing without a pause, compared to modes with a pause. The highest rate of increase in the interlayer temperature is observed when the first layers are deposited near the substrate. The presence of a pause leads to the fact that the temperature rises rapidly to around 10 layers, after which it changes insignificantly. However, the rapid increase in temperature continues after the 10th layer and almost stops after the 25th layer in the non-pause mode. The relative difference in the interlayer temperature in comparison with experimental points in all modes and at various distances from the substrate does not exceed 15% in absolute value. At the same time, deviations are observed both in the direction of overestimated values and in the direction of underestimated values.

**Figure 11:**
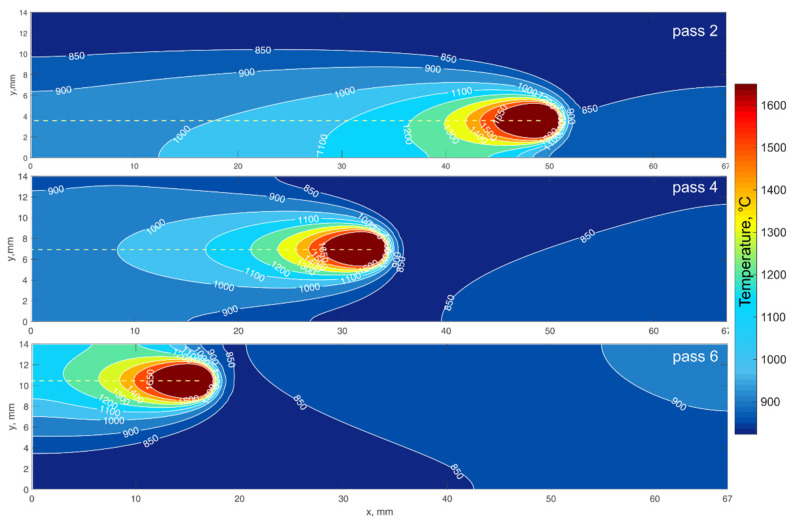
The calculated distribution of the wall surface temperature during the depositing of 2nd, 4th, and 6th passes of the 16th layer.

Figure 11 shows the calculated distribution of the wall surface temperature during the depositing of the second, fourth, and sixth passes of the 16th layer. It can be seen that the closer the melt pool is located to the side surface of the wall, the more the isotherms elongate. More elongated isotherms, other factors being equal, correspond to a lower cooling rate. This may cause the appearance of heterogeneity of grain sizes and properties along the wall width.

It should be noted once again that since the specificity of additive manufacturing technologies presupposes repetitive heating and cooling processes, ignoring convective heat transfer with the environment leads to a significant error. In addition, the time spent on the above calculations is on the order of several minutes or several tens of minutes using a personal computer. However, functional–analytical methods make it possible to obtain the value of a function at a certain moment in time; without calculating the values of the function in previous moments, the calculations are seconds.

## 5. Conclusions

The three-dimensional transient heat transfer model was developed for the DED processes of multi-track walls. The proposed model makes it possible to reproduce temperature fields at various values of the technological process parameters. This model is based on the obtained three-dimensional solution of the non-stationary heat conduction equation for a moving distributed heat source, considering convective heat transfer to the environment. Since the model made it possible to calculate the temperature change both in space and in time, the model was allowed to calculate all the temperature field characteristics (heat cycles, temperature gradients, and cooling rates). In this case, the wall and the substrate sizes, the change in power from layer to layer, the change in the cladding speed, the interpass dwell time, and the heat source trajectory were taken into account.

The relative difference in the interlayer temperature fluctuated within 15% when comparing the experimental and calculated data. The temperature change over time at fixed points of the samples was completely consistent with the experimental data. A decrease in temperature at a fixed point, as in the case of a mode without a pause, indicates the presence of a temperature gradient along the height of the wall, while it is practically absent with a pause of 25 s. According to calculations, the highest cooling rate is observed in the mode with the longest pause. Thus, the heating and cooling rate is proportional to the value of the pause time between passes and, consequently, inversely proportional to the values of interpass temperature.

Considering the features of beam or non-beam heat sources, it is possible to adapt the model to various direct deposition technologies. In addition, based on the proposed calculation scheme, it is possible to develop a method that takes into account any curved trajectories of the heat source.

## Figures and Tables

**Table 1:** Heating mode parameters.

Parameters		Passes									
		№1	№2	№3	№4	№5	№6	№7	№8	№9	№10
Mode 1	Power (W)	1000									
	Speed (mm/s)	10									
	Pause time (s)	5									
	Energy (kJ)	200									
Mode 2	Power (W)	1000									
	Speed (mm/s)	6		8		10		16		22	
	Pause time (s)	1	2	3	4	5	6	7	8	9	10
	Energy (kJ)	200									
Mode 3	Power (W)	450	500	550	600	650	700	800	850	900	950
	Speed (mm/s)	3	4	5	6	7	8	9	10	11	12
	Pause time (s)	0									
	Energy (kJ)	200									

**Table 2:** Deposition mode parameters.

Parameters	Value		
	Mode 1	Mode 2	Mode 3
Laser power (W)	1900	1900	1900
Cladding speed (inner bead) (mm/s)	20	20	20
Cladding speed (outer bead) (mm/s)	15	15	15
Laser spot diameter (mm)	2.5	2.5	2.5
Width offset (mm)	1.67	1.67	1.67
Height offset (mm)	0.8	0.8	0.8
Powder flow rate (g/min)	10.5	10.5	10.5
Pause time (s)	0	10	25

## Data Availability

Not applicable.

## References

[1] Uriondo A., Esperon-Miguez M., Perinpanayagam S. (2015). The Present and Future of Additive Manufacturing in the Aerospace Sector: A Review of Important Aspects. Proc. Inst. Mech. Eng. Part G J. Aerosp. Eng..

[2] Gisario A., Kazarian M., Martina F., Mehrpouya M. (2019). Metal Additive Manufacturing in the Commercial Aviation Industry: A Review. J. Manuf. Syst..

[3] Ahn D.G. (2016). Direct Metal Additive Manufacturing Processes and Their Sustainable Applications for Green Technology: A Review. Int. J. Precis. Eng. Manuf.-Green Technol..

[4] Korsmik R., Tsybulskiy I., Rodionov A., Klimova-Korsmik O., Gogolukhina M., Ivanov S., Zadykyan G., Mendagaliev R. (2020). The Approaches to Design and Manufacturing of Large-Sized Marine Machinery Parts by Direct Laser Deposition. Proc. Procedia CIRP.

[5] Turichin G.A., Klimova O.G., Zemlyakov E.V., Babkin K.D., Kolodyazhnyy D.Y., Shamray F.A., Travyanov A.Y., Petrovskiy P.V. (2015). Technological Aspects of High Speed Direct Laser Deposition Based on Heterophase Powder Metallurgy. Phys. Procedia.

[6] Ding D., Pan Z., Cuiuri D., Li H. (2015). Wire-Feed Additive Manufacturing of Metal Components: Technologies, Developments and Future Interests. Int. J. Adv. Manuf. Technol..

[7] Dhinakaran V., Stalin B., Ravichandran M., Balasubramanian M., Anand Chairman C., Pritima D. Wire Arc Additive Manufacturing Perspectives and Recent Developments. Proceedings of the IOP Conference Series: Materials Science and Engineering.

[8] Svetlizky D., Das M., Zheng B., Vyatskikh A.L., Bose S., Bandyopadhyay A., Schoenung J.M., Lavernia E.J., Eliaz N. (2021). Directed Energy Deposition (DED) Additive Manufacturing: Physical Characteristics, Defects, Challenges and Applications. Mater. Today.

[9] Gushchina M.O., Yu Ivanov S., Vildanov A.M. Effect of Temperature Field on Mechanical Properties of Direct Laser Deposited Ti-6Al-4V Alloy. Proceedings of the IOP Conference Series: Materials Science and Engineering.

[10] Bambach M., Sizova I., Szyndler J., Bennett J., Hyatt G., Cao J., Papke T., Merklein M. (2021). On the Hot Deformation Behavior of Ti-6Al-4V Made by Additive Manufacturing. J. Mater. Process. Technol..

[11] Saboori A., Abdi A., Fatemi S.A., Marchese G., Biamino S., Mirzadeh H. (2020). Hot Deformation Behavior and Flow Stress Modeling of Ti–6Al–4V Alloy Produced via Electron Beam Melting Additive Manufacturing Technology in Single β-Phase Field. Mater. Sci. Eng. A.

[12] Ivanov S., Gushchina M., Artinov A., Khomutov M., Zemlyakov E. (2021). Effect of Elevated Temperatures on the Mechanical Properties of a Direct Laser Deposited Ti-6Al-4V. Materials.

[13] Klimova-Korsmik O.G., Turichin G.A., Shalnova S.A., Gushchina M.O., Cheverikin V.V. (2018). Structure and Properties of Ti-6Al-4V Titanium Alloy Products Obtained by Direct Laser Deposition and Subsequent Heat Treatment. J. Phys. Conf. Ser..

[14] Kelly S.M., Kampe S.L. (2004). Microstructural Evolution in Laser-Deposited Multilayer Ti-6Al-4V Builds: Part 1. Microstructural Characterization. Metall. Mater. Trans. A Phys. Metall. Mater. Sci..

[15] Lia F., Park J.Z., Keist J.S., Joshi S., Martukanitz R.P. (2018). Thermal and Microstructural Analysis of Laser-Based Directed Energy Deposition for Ti-6Al-4V and Inconel 625 Deposits. Mater. Sci. Eng. A.

[16] Vildanov A.M., Babkin K.D., Alekseeva E.V. (2020). Macro Defects in Direct Laser Deposition Process. Mater. Today Proc..

[17] Rodriguez E., Mireles J., Terrazas C.A., Espalin D., Perez M.A., Wicker R.B. (2015). Approximation of Absolute Surface Temperature Measurements of Powder Bed Fusion Additive Manufacturing Technology Using in Situ Infrared Thermography. Addit. Manuf..

[18] Tang Z.J., Liu W.W., Wang Y.W., Saleheen K.M., Liu Z.C., Peng S.T., Zhang Z., Zhang H.C. (2020). A Review on in Situ Monitoring Technology for Directed Energy Deposition of Metals. Int. J. Adv. Manuf. Technol..

[19] Yang D., Wang G., Zhang G. (2017). Thermal Analysis for Single-Pass Multi-Layer GMAW Based Additive Manufacturing Using Infrared Thermography. J. Mater. Process. Technol..

[20] Schöpp H., Sperl A., Kozakov R., Gött G., Uhrlandt D., Wilhelm G. (2012). Temperature and Emissivity Determination of Liquid Steel S235. J. Phys. D Appl. Phys..

[21] Lane B., Moylan S., Whitenton E.P., Ma L. (2016). Thermographic Measurements of the Commercial Laser Powder Bed Fusion Process at NIST. Rapid Prototyp. J..

[22] Peyre P., Dal M., Pouzet S., Castelnau O. (2017). Simplified Numerical Model for the Laser Metal Deposition Additive Manufacturing Process. J. Laser Appl..

[23] Kiran A., Hodek J., Vavřík J., Urbánek M., Džugan J. (2020). Numerical Simulation Development and Computational Optimization for Directed Energy Deposition Additive Manufacturing Process. Materials.

[24] Wang L., Felicelli S., Gooroochurn Y., Wang P.T., Horstemeyer M.F. (2008). Optimization of the LENS^®^ Process for Steady Molten Pool Size. Mater. Sci. Eng. A.

[25] Malmelöv A., Lundbäck A., Lindgren L.E. (2020). History Reduction by Lumping for Time-Efficient Simulation of Additive Manufacturing. Metals.

[26] Michaleris P. (2014). Modeling Metal Deposition in Heat Transfer Analyses of Additive Manufacturing Processes. Finite Elem. Anal. Des..

[27] Peyre P., Aubry P., Fabbro R., Neveu R., Longuet A. (2008). Analytical and Numerical Modelling of the Direct Metal Deposition Laser Process. J. Phys. D. Appl. Phys..

[28] Heigel J.C., Michaleris P., Reutzel E.W. (2015). Thermo-Mechanical Model Development and Validation of Directed Energy Deposition Additive Manufacturing of Ti-6Al-4V. Addit. Manuf..

[29] Chiumenti M., Lin X., Cervera M., Lei W., Zheng Y., Huang W. (2017). Numerical Simulation and Experimental Calibration of Additive Manufacturing by Blown Powder Technology. Part I: Thermal Analysis. Rapid Prototyp. J..

[30] Denlinger E.R., Irwin J., Michaleris P. (2014). Thermomechanical Modeling of Additive Manufacturing Large Parts. J. Manuf. Sci. Eng. Trans. ASME.

[31] Patil N., Pal D., Rafi H.K., Zeng K., Moreland A., Hicks A., Beeler D., Stucker B. (2015). A Generalized Feed Forward Dynamic Adaptive Mesh Refinement and Derefinement Finite Element Framework for Metal Laser Sintering—Part I: Formulation and Algorithm Development. J. Manuf. Sci. Eng. Trans. ASME.

[32] Ou W., Knapp G.L., Mukherjee T., Wei Y., DebRoy T. (2021). An Improved Heat Transfer and Fluid Flow Model of Wire-Arc Additive Manufacturing. Int. J. Heat Mass Transf..

[33] Manvatkar V., De A., DebRoy T. (2015). Spatial Variation of Melt Pool Geometry, Peak Temperature and Solidification Parameters during Laser Assisted Additive Manufacturing Process. Mater. Sci. Technol..

[34] Gan Z., Liu H., Li S., He X., Yu G. (2017). Modeling of Thermal Behavior and Mass Transport in Multi-Layer Laser Additive Manufacturing of Ni-Based Alloy on Cast Iron. Int. J. Heat Mass Transf..

[35] Li J., Wang Q., Michaleris P., Reutzel E.W., Nassar A.R. (2017). An Extended Lumped-Parameter Model of Melt-Pool Geometry to Predict Part Height for Directed Energy Deposition. J. Manuf. Sci. Eng. Trans. ASME.

[36] Li J., Wang Q., Michaleris P. (2018). An Analytical Computation of Temperature Field Evolved in Directed Energy Deposition. J. Manuf. Sci. Eng. Trans. ASME.

[37] Huang Y., Khamesee M.B., Toyserkani E. (2019). A New Physics-Based Model for Laser Directed Energy Deposition (Powder-Fed Additive Manufacturing): From Single-Track to Multi-Track and Multi-Layer. Opt. Laser Technol..

[38] Mukin D., Valdaytseva E., Turichin G. (2021). Analytical Solution of the Non-Stationary Heat Conduction Problem in Thin-Walled Products during the Additive Manufacturing Process. Materials.

[39] Howlett J., Abramowitz M., Stegun I.A. (1966). Handbook of Mathematical Functions. Math. Gaz..

[40] Mills K.C. (2002). Recommended Values of Thermophysical Properties for Selected Commercial Alloys.

